# Product- and schedule-specific vaccine effectiveness against invasive *Haemophilus influenzae* serotype b (Hib) disease, The Netherlands, 2005 to 2023

**DOI:** 10.2807/1560-7917.ES.2026.31.19.2500699

**Published:** 2026-05-14

**Authors:** Marta Bertran, Brechje de Gier, Tatiana Garcia Vilaplana, Nina M van Sorge, Hester E de Melker, Anneke Steens

**Affiliations:** 1 Centre for Infectious Disease Control, National Institute for Public Health and the Environment (RIVM), Bilthoven, The Netherlands; 2 European Programme for Intervention Epidemiology Training (EPIET), European Centre for Disease Prevention and Control (ECDC), Stockholm, Sweden; 3 Department of Medical Microbiology and Infection Prevention, Amsterdam Institute for Immunology and Infectious Diseases, Amsterdam UMC, University of Amsterdam, Amsterdam, The Netherlands; 4 Netherlands Reference Laboratory for Bacterial Meningitis (NRLBM), Amsterdam UMC, AMC, Amsterdam, The Netherlands

**Keywords:** Case-control, NIP, Immunization programme, Hexavalent, 2+1, vaccination, vaccine effectiveness, Haemophilus, Hib

## Abstract

**BACKGROUND:**

Invasive *Haemophilus influenzae* type b (iHib) disease incidence in < 5-year-olds decreased after vaccine introduction but increased between 2011 and 2023 in the Netherlands. The National Immunisation Programme changed products in 2011 (from DTaP-IPV/Hib to DT3aP-HBV-IPV/Hib) and 2018 (to DT5aP-HBV-IPV-Hib) and schedule from 3 + 1 to 2 + 1 doses in 2020.

**AIM:**

We aimed to estimate overall, product- and schedule-specific vaccine effectiveness (VE) against iHib disease to inform vaccination strategies.

**METHODS:**

We conducted a matched case–control study extracting iHib cases born ≥ 2005 and aged 6–119 months from 2005–2023 national reference laboratory data. We selected 10 controls per case matched on birth date and sex from the population register and obtained vaccination data from the vaccination registry. Using conditional logistic regression, we estimated matched odds ratios (mOR) and VE among 6–10-month-olds (eligible only for the primary series) and 11–119-month-olds.

**RESULTS:**

We included 250 iHib cases and 2,487 controls. Among children aged 11–119 months, VE against iHib of the full schedule was 96% (95% CI: 88–99), 95% (95% CI: 91–97) and 98% (95% CI: 94–99) for any DTaP-IPV/Hib, DT3aP-HBV-IPV/Hib and DT5aP-HBV-IPV-Hib, respectively. It was 97% (95% CI: 93–99) for 2 + 1, 96% (95% CI: 93–98) for 3 + 1 doses, and > 95% for the respective primary series. No differences in VE by time since vaccination were observed between products or schedules.

**CONCLUSION:**

Changes in VE against iHib after recent product or schedule changes do not explain the increasing iHib incidence. The high VE supports pursuing optimal vaccination coverage.

**Key public health message**

**What did you want to address in this study and why?**

Serious infections caused by *Haemophilus influenzae* type b (Hib) in young children have increased in the Netherlands over the last decade. During this time, the vaccines used, the number of doses, and the timing of childhood vaccinations also changed. We wanted to understand whether these changes affected individual protection against Hib disease, by comparing the different vaccines and vaccination schedules.

**What have we learnt from this study?**

The different products and schedules used in the Netherlands between 2005 and 2023 provided equal protection of young children against Hib disease. Protection was higher in the first year after completing the vaccination schedule compared with 1–3 years after, but there were no differences between vaccines or schedules. This suggests that the rise in serious Hib infections is not because the vaccines have become less effective.

**What are the implications of your findings for public health?**

Public health efforts should continue to focus on vaccination coverage, as vaccination remains highly effective against Hib disease.

## Introduction

The bacterium *Haemophilus influenzae* (Hi) can be carried asymptomatically in the nasopharynx or cause invasive diseases such as meningitis and pneumonia. It is classified as non-encapsulated or encapsulated, discriminating six serotypes (a-f). Following introduction of Hi serotype b (Hib) conjugate vaccines in the 1990s, invasive Hib disease (iHib) incidence dropped considerably [1]. In the Netherlands, vaccination was introduced in 1993 at 3, 4, 5 and 11 months of age. In 1999, the schedule changed to 2, 3, 4 and 11 months [2]. Different combination vaccines were subsequently introduced: a pentavalent vaccine combined Hib with diphtheria, tetanus, whole-cell pertussis (DTwP) and inactivated polio vaccine (IPV) (DTwP-IPV/Hib) in 2003, replaced by pentavalent vaccines with an acellular pertussis component in 2005 (DTaP-IPV/Hib). In 2006, a hexavalent vaccine containing an additional hepatitis B virus (HBV) component (DT3aP-HBV-IPV/Hib) was introduced for high-risk children and extended to all children in 2011 [2]. Details of the products and schedules used in the National Immunisation Programme (NIP) are appended in Supplementary Table S1. 

After the highest coverage in 2-year-olds for Hib-containing vaccines was observed in 2012 (96.1%), the coverage decreased but remained above 90% up to 2021 [3]. Nevertheless, iHib incidence in < 5-year-olds has been slowly increasing since 2011 (from ca 0.7/100,000 to a peak of 3.3/100,000 in 2022) [2]. A potential explanation was a decrease in vaccine effectiveness (VE) associated with the change from pentavalent to hexavalent vaccines in 2011. A matched case–control study by our group covering the years 2003 to 2016 estimated a VE of 93% (95% confidence interval (CI): 89–95) against iHib in < 5-year-olds, without differences in VE between the hexavalent and pentavalent vaccines in any age group [4].

Since this previous VE study, two new changes related to Hib-containing vaccines occurred in the Dutch NIP. Firstly, there was a product change from DT3aP-HBV-IPV/Hib (henceforth Hexavalent I) to DT5aP-HBV-IPV-Hib (Hexavalent II) for children born from October 2018. These products contain different carrier compounds, adjuvants and lengths of the Hib component and a different number of pertussis antigens [5]. Secondly, in 2020 the schedule changed from a 3 + 1 (2, 3, 4 and 11 months) to a 2 + 1 (3, 5 and 11 months) schedule, unless the mother had not received maternal pertussis vaccination or the child had risk factors (e.g. prematurity, maternal immunosuppression), in which case a 3 + 1 schedule at 2, 3, 5 and 11 months is offered [2,6].

Meanwhile, the increasing trend in iHib incidence among < 5-year-olds continued up to 2022, with an even sharper increase in 2020, when other invasive bacterial diseases and non-b Hi disease decreased in the Netherlands and globally as a result of the measures taken in response to the COVID-19 pandemic [2,7-9].

A lower VE for the recently introduced product (Hexavalent II) or schedule (2 + 1) remained as potential explanations for the continued high iHib incidence in < 5-year-olds, and the unexpected iHib increase in the Netherlands during the COVID-19 pandemic. The VE estimates using the screening method remained above 90% in 2020 (97%; 95% CI: 93–99) and 2021 (91%; 95% CI: 78–97), similar to the period 2015 to 2019 (92%; 95% CI: 88–95) [7]. An age-specific analysis indicated that VE using the screening method remained ≥ 90% in 7–11-month-olds and 1-year-olds in the periods 2008 to 2012, 2013 to 2017 and 2018 to 2022, but decreased in 2-year-olds from ≥ 90% in 2008 to 2012 and 2013 to 2017 to 84% in 2018 to 2022 [10]. However, none of these analyses were product- or schedule-specific. Also, these screening method estimates relied on limited and partly self-reported vaccination data rather than linked vaccination registry data.

Here, we aimed to estimate overall, product- and schedule-specific VE against iHib in the Netherlands in individuals born in 2005 or later and aged 6–119 months during the period 2005 to 2023. In particular, we aimed to determine whether lower VE of the latest NIP product or the 2 + 1 schedule could have contributed to the increasing incidence.

## Methods

### Study design

We conducted a matched case–control study including, as cases, persons born 2005 or later who developed iHib between 1 January 2005 and 31 December 2023 and were aged 6–119 months (i.e. < 10 years) at the time of disease based on the surveillance dataset of the Netherlands Reference Laboratory for Bacterial Meningitis (NRLBM). Controls were selected from the population registry, matched on birth date (± 1 day) and sex to include 10 controls per case. Cases and controls were linked to the national childhood vaccination registry (Præventis) using birth date, sex, name and 6-digit postcode. After pseudonymisation of identifiable data and medical data separately by a trusted third party, linkage was conducted so that no individual had access to linked identifiable and medical information. Sex, postcode and name were removed from the data accessed by the researchers. Cases and controls who could not be linked to Præventis or controls who were not in the population registry at the case index date (due to death/emigration) were excluded. Excluded controls were not replaced. Therefore, some cases had fewer than 10 controls matched. We included only the first iHib episode per case.

### Definitions

iHib was defined as isolation of Hib in a normally sterile site (e.g. blood, cerebrospinal fluid or other body fluids). Laboratories in the Netherlands are requested to send invasive Hi isolates to the NRLBM for serotyping, which is done by co-agglutination with Hib-specific antibodies coupled to *Staphylococcus aureus* protein A.

We defined index date as the sample date for cases where the interval between sample date and the date of receipt by the NRLBM was shorter than 38 days. We used the median interval from these cases to estimate the index date in cases where this interval was longer than 37 days or with an unknown sample date, by calculating the NRLBM receipt date minus the median. For controls, we assigned the index date of their corresponding case. Vaccination status was defined as fully vaccinated, partially vaccinated and unvaccinated based on any vaccine received 14 or more days before the index date. Children younger than 1 year with two or more doses received at least 2 weeks apart, or children 1 year or older, who had received a booster dose or any dose from 12 months of age, were considered fully vaccinated. Unvaccinated included only children with 0 doses registered. The remaining were partially vaccinated. A booster dose was any dose at least 120 days after the primary series for children born before 2020 or at least 150 days for children born in 2020 or later, in line with national guidelines. Vaccine failures were fully vaccinated cases.

The vaccine product was defined based on the last product received before the index date and categorised as any pentavalent (DT3aP-IPV/Hib), Hexavalent I (DT3aP-HBV-IPV/Hib), Hexavalent II (DT5aP-HBV-IPV-Hib) and other/unknown for the main analysis. We combined the different pentavalent vaccines used in the NIP because between 2007 and 2009 these vaccines were used interchangeably [2]. In a sensitivity analysis, we defined the product based on all doses; where the product was known for more than one dose but differed, we classified it as mixed. The unknown category included only children for whom all doses were unknown.

We classified the vaccination schedule as 3 + 1 or 2 + 1 based on the number of doses received as the primary series (three or two). For children who only received the primary series, we classified it as 3 + 0 or 2 + 0. Participants who had received vaccine doses in other schedules (e.g. 4 + 1 or 1 + 1) could be included as ‘fully vaccinated’ in the overall VE analysis but were excluded from the product- and schedule-specific analyses. We also categorised children by vaccine eligibility based on age: 6–10 months (eligible for primary series) and ≥ 11 months (eligible for full schedule). To ensure that cases and controls were assigned the same age category, we assigned controls the birth date of their matched case. We defined time since vaccination (TSV) as the interval between the booster dose and the index date and categorised it as < 1 year, 1 year to < 4 years and ≥ 4 years (with a maximum of 9 years).

### Statistical analyses

For categorical variables, we calculated proportions. For continuous variables (age and TSV), we calculated medians and inter-quartile ranges (IQR). Using conditional logistic regression, we estimated matched odds ratios (mOR) comparing fully vaccinated to unvaccinated children, where each group (case + matched controls) was considered a stratum. No further covariates were included in the model. We calculated VE as VE = 1 − mOR and estimated overall, product- and schedule-specific VE for children eligible for the full schedule (≥ 11-month-olds), the primary series only (6–10-month-olds) during the whole period and by TSV. Overall, VE estimates compared unvaccinated children against all children fully vaccinated according to the NIP. For product- and schedule-specific estimates, only children having received 2 + 0/3 + 0 (6–10-month-olds) and 2 + 1/3 + 1 (≥ 11-month-olds) schedules were included and compared with unvaccinated children. For product- and schedule-specific VE, we excluded participants with TSV ≥ 4 years to ensure comparability between products and schedules, as the most recently introduced product (Hexavalent II) and schedule (2 + 1) have not been used long enough for longer follow-up. We considered VE estimates different if 95% CIs did not overlap, but for pairs with minimally overlapping 95% CIs, we compared estimates using a Wald test and considered it significant at p < 0.05 [11].

## Results

Between 1 January 2005 and 31 December 2023, 259 Hib cases among persons who were born 2005 or later and were older than 6 months or younger than 119 months at the index date were reported in the Netherlands, accounting for 35% (n = 750) of all Hib cases in this period. After excluding six cases that could not be linked to Præventis, three second episodes, and three controls that were also cases, we included 250 Hib cases and 2,487 controls in the study. The number of included cases increased steadily over time, from two cases in 2005 to a maximum of 29 cases in 2020 and 2022, partly explained by the expanding study population as more birth cohorts were included each year. Cases had a median age at infection of 19 months (IQR: 10–35).

Among cases, 53% (133/250) were fully vaccinated, compared with 93% (2,318/2,487) controls, while the proportion of partially vaccinated was < 3% in both cases and controls (Table).

**Table:** Characteristics of invasive *Haemophilus influenzae* serotype b disease cases and matched controls included in the case–control study, the Netherlands, 2005–2023 (n = 2,737)

Category	Overall n = 2,737		Cases n = 250		Controls n = 2,487	
	n	%	n	%	n	%
Age group						
6–10 months	811	30	74	30	737	30
11–23 months	752	27	69	28	683	27
2 to < 3 years	493	18	45	18	448	18
3 to < 5 years	450	16	41	16	409	16
5 to < 10 years	231	8.4	21	8.4	210	8.4
Birth cohort						
2005–2007	219	8.0	20	8.0	199	8.0
2008–2010	381	14	35	14	346	14
2011–2013	438	16	40	16	398	16
2014–2016	494	18	45	18	449	18
2017–2019	701	26	64	26	637	26
2020–2023	504	18	46	18	458	18
Vaccination status						
Unvaccinated	214	7.8	113	45	101	4.1
Fully vaccinated	2,451	90	133	53	2,318	93
Partially vaccinated	72	2.6	4	1.6	68	2.7
Vaccination schedule^a^						
2 + 0	186	7.4	9	6.6	177	7.4
2 + 1	237	9.4	10	7.3	227	9.5
3 + 0	596	24	31	23	565	24
3 + 1	1,460	58	86	63	1,374	58
Other	44	1.7	1	0.7	43	1.8
Vaccination product last dose^a^						
Any pentavalent	509	20	36	26	473	20
Hexavalent I	1,190	47	68	50	1,122	47
Hexavalent II	767	30	30	22	737	31
Unknown/other	57	2.3	3	2.2	54	2.3

^a^ Vaccination status and schedule is only included for those who received at least one dose (n = 2,523).

**Figure 1:**
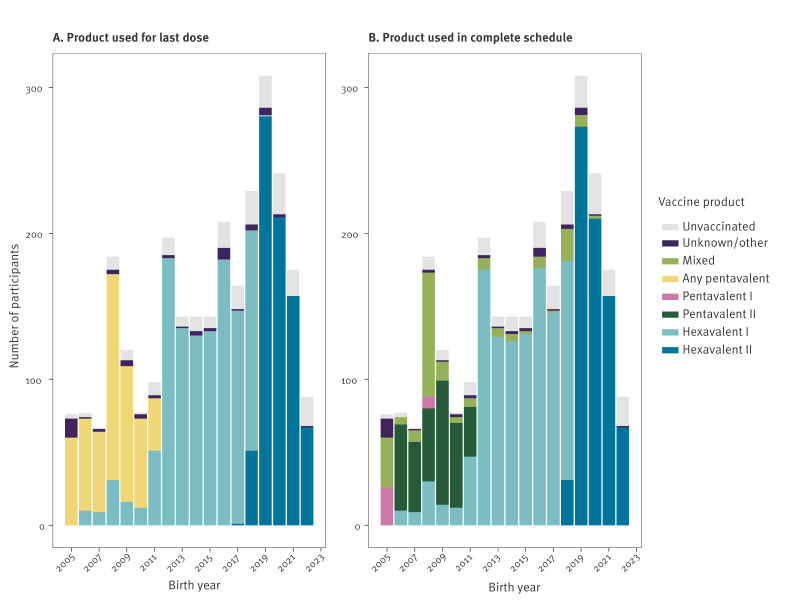
*Haemophilus influenzae* serotype b-containing vaccine product received by study participants, by birth year, the Netherlands, 2005–2023 (n = 2,737)

Information on the last vaccine product received was available for 98% of participants. Hexavalent I was received by 1,190 (47%) of the 2,523 vaccinated participants (Table), of whom 97% (1,155/1,190) received it for all doses. Similarly, among 767 (30%) of vaccinated children who received Hexavalent II as the last product, 96% received it for all doses (n = 738) (Figure 1). For comparison, among children who received any pentavalent as their last dose, 72% (368/509) received the same product for all doses. The products used by birth year mostly aligned with the NIP recommendations (Figure 1). The vaccine products received by participants by index year are appended in Supplementary Figure S1. 

**Figure 2:**
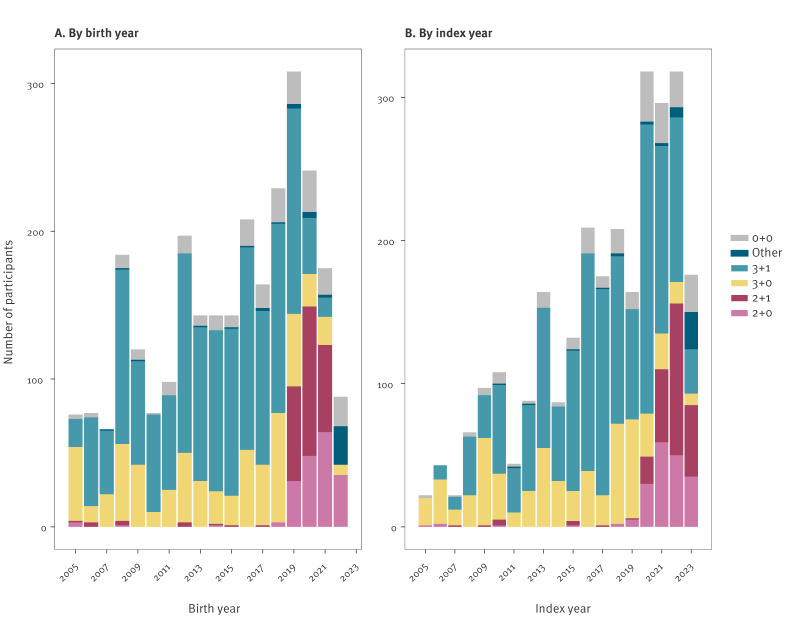
*Haemophilus influenzae* serotype b vaccination schedule of participants, the Netherlands, 2005–2023 (n = 2,737)

Among participants who received at least 1 dose, 58% received a 3 + 1 schedule, followed by a 3 + 0 schedule (24%). Of these, 95% (n = 1,957) were born before 2020, when the standard NIP schedule was 3 + 1 doses (Figure 2). The received schedule was similar between vaccinated cases and controls (Table). Cases had a longer median TSV (544 days; IQR: 193–1,007) than controls (264 days; IQR: 141–714). Within 1–3 years since vaccination, median TSV was shorter for Hexavalent II and the 2 + 1 schedule than for previous products and for the 3 + 1 schedule. A breakdown of the median TSV for each TSV category, overall and by product and schedule, is appended in Supplementary Tables S2 and S3. 

**Figure 3:**
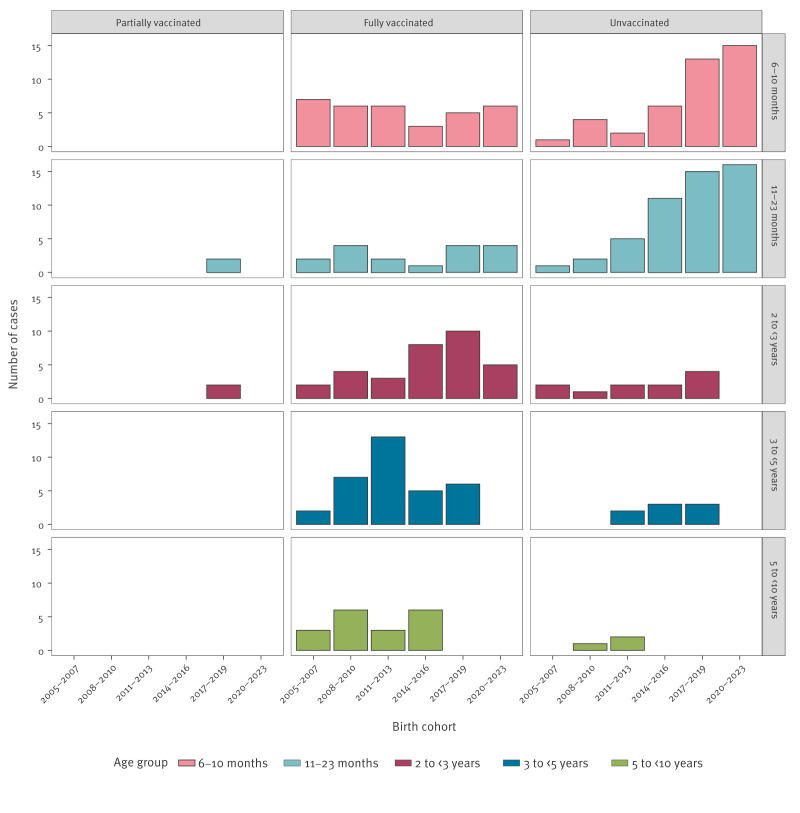
Number of invasive *Haemophilus influenzae* serotype b cases by age group, vaccination status, and birth cohort, the Netherlands, 2005–2023 (n = 250)

Among the 133 vaccine failures, 115 (86%) were in children between the age of 6 months and < 5 years. Only 13% (n = 17) of vaccine failures were in 11–23-month-olds, an age group that accounted for 44% (n = 50) of unvaccinated cases. (Figure 3).

**Figure 4:**
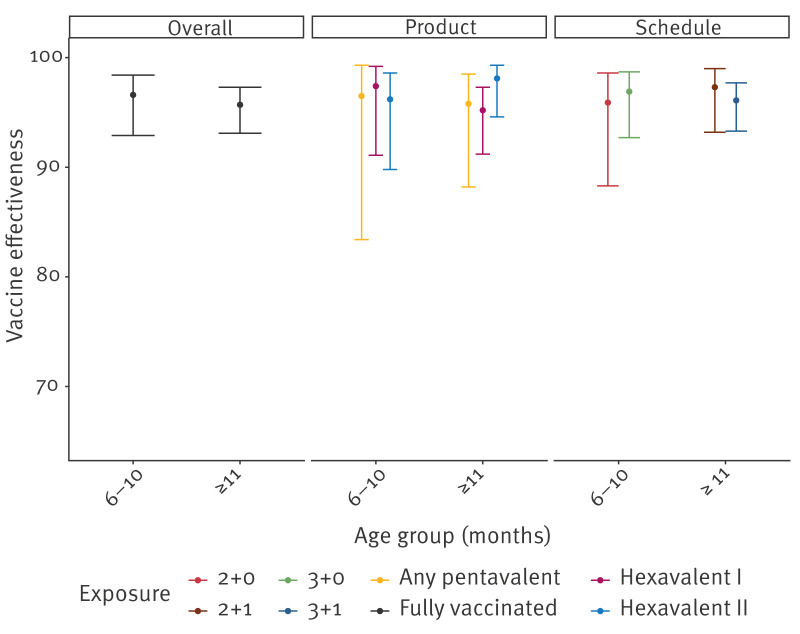
Overall, product- and schedule-specific vaccine effectiveness against invasive *Haemophilus influenzae* serotype b disease by age group, the Netherlands, 2005–2023 (n = 2,665)

Overall (product-independent) VE was similar after the primary series (6–10-month-olds: 96.6%; 95% CI: 92.9–98.4) and for 11–119-month-olds (95.7%; 95% CI: 93.1–97.3) (Figure 4). Among 11–59-month-olds, overall VE was 96.1% (95% CI: 93.6–97.6). The VE for Hexavalent II was above 96% in 6–10-month-olds and those ≥ 11-month-olds who had received a booster (Figure 4). Generally, the product-specific VEs were not different from each other for any age group, as the 95% CIs overlapped, with point estimates ≥ 95% (range: 95–98%) (Figure 4). There was one exception, which was a lower VE the first year after the booster for any pentavalent vaccine compared with both Hexavalent I and II (Wald test: p < 0.02), however, this difference disappeared a year after vaccination. By schedule, VE was similar for the 2 + 1 (97.3%; 95% CI: 93.2–99.0), and the 3 + 1 schedule (96.1%; 95% CI: 93.3–97.7), after the booster (≥ 11-month-olds; Figure 4). Furthermore, there were no differences in VE for the primary series by schedule (2 + 0 vs 3 + 0) among 6–10-month-olds. VE estimates and underlying numbers for all strata are appended in Supplementary Table S4.

Whiskers represent 95% confidence intervals. Vaccine effectiveness estimates compared fully vaccinated with unvaccinated children, partially vaccinated are excluded.

**Figure 5:**
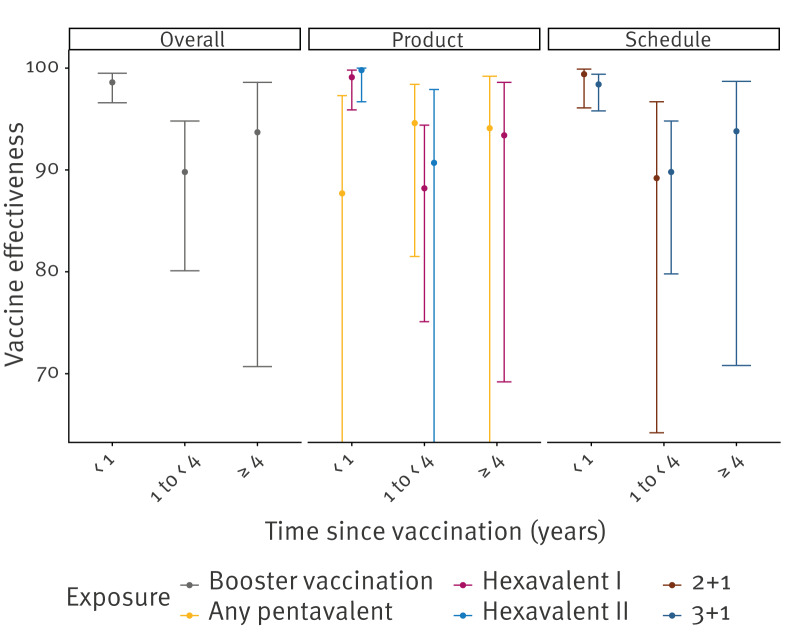
Overall, product- and schedule-specific vaccine effectiveness against invasive *Haemophilus influenzae* serotype b disease by time since vaccination in children aged ≥ 11 months who received the booster dose, the Netherlands, 2005–2023 (n =1,883)

After the booster (11–119-month-olds), VE decreased by TSV from 98.6% (95% CI: 96.6–99.5) in the first year to 89.8% (95% CI: 80.1–94.8) between 1 year and < 4 years (Figure 5). Vaccine effectiveness estimates for ≥ 4 years since vaccination were very uncertain. We observed no differences by TSV between products or schedules (Figure 5).

Whiskers represent 95% confidence intervals. Vaccine effectiveness estimates compared fully vaccinated with unvaccinated children, partially vaccinated are excluded.

In a sensitivity analysis considering the product used in the complete schedule, VE was similar for both hexavalent vaccines and similar to the VE based on the last product received, probably reflecting the fact that most children received the same hexavalent vaccine for all doses. However, in the first year after the booster, the VE for the mixed schedule was lower than the VE of both hexavalent products (p < 0.015). We observed no differences for the different pentavalent vaccines, but 95% CIs were wide. All estimates for this sensitivity analysis are appended in Supplementary Table S5.

## Discussion

Our study found high VE against iHib across the different vaccine products and schedules used in the Dutch NIP, both in children aged 11–119 months eligible for the full schedule and in 6–10 months-old infants who had only been eligible for the primary series. The VE was not different between products or schedules at least up to 4 years after vaccination. While surveillance data based on self-reported and incomplete data did not imply an increasing incidence among fully vaccinated children, our results using linked vaccination registry data confirm that the sustained VE against iHib for the latest product (Hexavalent II) or schedule (2 + 1) used in the NIP did not contribute to the additional increase in iHib cases in the Netherlands between 2020 to 2023.

These results are in line with VE estimates for the period 2015 to 2021 obtained through the screening method, where no differences were observed in 2020 and 2021 compared with the VE between 2015 and 2019 [7]. A previous product-specific case–control study also suggested high VE and no difference between the Hexavalent I and pentavalent vaccines, nor a decrease over time [4]. A meta-analysis using case–control studies in settings with different schedules and products estimated VE against iHib at 92% and 95% for two and three doses, respectively, resembling our estimates [12]. In other studies, VE ranged from 89 to 99%, with an outlier from the United Kingdom (VE = 57%), attributed, among other factors, to the lack of a booster dose and use of DTaP-Hib (acellular pertussis) instead of the DTwP-Hib (whole-cell pertussis) [12-14].

Immunogenicity and vaccine effectiveness (VE) are not equivalent; however, antibody responses are often used as a proxy indicator of potential protection. Geometric mean concentrations (GMC) reflect the average antibody level elicited in a population, with higher GMCs generally indicating a stronger humoral immune response and potentially greater protection. Based on the higher GMCs observed in immunogenicity studies, one might have expected higher VE after the booster dose with Hexavalent I compared with Hexavalent II. Previous immunogenicity studies indicated higher antibody levels (i.e. IgG anti-polyribosylribitol phosphate (PRP) GMC) after the booster dose with Hexavalent I compared with Hexavalent II administered as a 2 + 1 or a 3 + 1 schedule. However, after the primary series and before the booster, anti-PRP GMC were lower with Hexavalent I [5]. The proportion of children with anti-PRP antibody concentrations associated with short-term (≥ 0.15 μg/mL) and long-term protection (≥ 1.0 μg/mL) followed a similar pattern [5]. The clinical significance of these differences, if any, has not been previously established. Our study does not provide evidence for clinical differences between products, or between the primary series and the full schedule. The VE among 6–10-month-olds was high and similar between products.

In France, where a 2 + 1 schedule with a start at slightly younger age (2, 4 and 11 months) than in the Netherlands has been used since 2013, an increase in iHib cases younger than 5 years was reported between 2017 and 2019, rising further in 2020 and 2021 [15,16]. Compared with children on the former French 3 + 1 schedule (2, 3, 4 months and 16–18 months), children younger than 4 years on the 2 + 1 schedule in France had lower anti-PRP IgG levels overall, and significantly lower in 2-year-olds. The proportion of children younger than 4 years achieving long-term protection (anti-PRP IgG ≥ 1.0 μg/mL) with the 2 + 1 (25%) was also lower compared with the 3 + 1 schedule (56%) [15]. While differences in 2-year-olds may partly reflect the shorter time since the booster in the 3 + 1 schedule, these findings align with previous immunogenicity studies [5] and may suggest decreased protection after the 2 + 1 versus the 3 + 1 schedule, which subsequently may have contributed to the observed increase in iHib incidence [15]. Still, Portugal, where a 3 + 1 schedule (2, 4, 6 and 18 months) is used, also described an increase in iHib incidence and vaccine failures in < 18-year-olds between 2016 and 2021 compared with the period 2010 to 2015. No risk factors for vaccine failures were identified [17]. To our knowledge, no other country has reported an iHib increase in recent years.

Although VE against iHib is high, lower immunogenicity from different vaccine products and schedules could plausibly lead to increased colonisation, and thus increased transmission and disease across the population regardless of vaccination status. Decreased protection against colonisation might explain the observed rise in incidence, potentially exacerbated by declining vaccine coverage [3]. While France reported increased iHib cases 5 years after implementing a 2 + 1 schedule, it is unlikely that the Netherlands’ 2 + 1 schedule implementation in 2020 caused an increase in the same year. Furthermore, other countries using a 2 + 1 schedule, such as Finland, Sweden and Spain have, to our knowledge, maintained stable iHib incidence [18,19].

As other Hib VE studies, our study only focused on iHib and cannot address possible changes in VE against transmission. A 2018 carriage study in the Netherlands did not identify any Hib among 300 2-year-olds [20]. However, given the low Hib carriage prevalence associated with Hib vaccination programmes [21,22], such studies may be insufficiently powered to detect Hib carriage changes. A 2016/17 seroprevalence study indicated that Hib GMC antibodies were slightly lower among 2–6-year-olds (eligible for Hexavalent I) compared with 6–20-year-olds (eligible for pentavalent vaccination) [20]. However, anti-PRP IgG seroprevalence decreased also in unvaccinated age groups between 2005/06 and 2016/17 [2], suggesting that decreased Hib exposure has reduced natural immunity. Ongoing seroprevalence studies may provide further insights into population protection following NIP changes.

The availability of individual level product- and schedule-specific data is a strength of this study, allowing us to estimate product- and schedule-specific VE. By matching on birth date, we minimised the confounding effect of age which affects disease risk and the vaccine and schedule received, as these are recommended by birth cohort.

A limitation in our study is the implementation of informed consent for registration of vaccinations from 2022, which may have caused exposure misclassification, as persons with unregistered vaccinations would appear as unvaccinated in our dataset. It is estimated that 2–3% of Hib-containing vaccinations were not registered [3]. This may have led to underestimation of VE among the youngest birth cohorts receiving Hexavalent II in the 2 + 1 schedule, regardless of whether misclassification occurred more often among cases, controls or non-differentially [23].

Despite the relatively high iHib incidence and the inclusion of 97% of all eligible cases during the study period, the Netherlands’ population size of ca 17 million during the study period limited statistical power and we may have missed a true difference in VE, as 95% CIs were large. To account for a shorter follow-up time for the latest product and schedule (Hexavalent II and 2 + 1), we limited product- and schedule-specific estimates to less than 4 years post-vaccination. However, the median TSV remained shorter for these than for Hexavalent I and 3 + 1, respectively, even within TSV categories. Power was particularly low for the 2 + 1 schedule, for which follow-up time and case numbers were low; further monitoring is warranted. Children who received the 2 + 1 schedule before its standard implementation in 2020 may have had a (medical) reason and may be different from the general population, but numbers were too small to restrict the analysis to children born 2020 or later. We lacked data on confounders such as comorbidities or prematurity, which influenced the NIP product/schedule recommendations and may affect infection risk and immune response. However, comorbidity prevalence in cases younger than 5 years was < 10% in 2023 [2]. Finally, comparisons between products and schedules should be interpreted with caution, as these correspond to specific time periods, and we were unable to correct for possible time-related confounders.

## Conclusion

We did not observe lower VE against invasive Hib disease for Hexavalent II and the 2 + 1 schedule compared with earlier products and schedules, suggesting that the observed iHib incidence could not be explained by lower VE following NIP changes in the Netherlands. The VE of the NIP against iHib disease remained high, reaffirming that a high vaccine coverage can minimise disease burden.

## Data Availability

Data cannot be shared on the individual level due to privacy reasons. Aggregated data are available upon reasonable request to the corresponding author.

## Supplementary Data

1337000 Supplement

## References

[1] Slack MPE Cripps AW Grimwood K Mackenzie GA Ulanova M. Invasive Haemophilus influenzae infections after 3 decades of Hib protein conjugate vaccine use. Clin Microbiol Rev. 2021;34(3):e0002821. 10.1128/CMR.00028-21 34076491 PMC8262803

[2] Rijksinstituut voor Volksgezondheid en Milieu (RIVM). The National Immunisation Programme in the Netherlands. Surveillance and developments in 2023-2024. Bilthoven: RIVM; 2024. Available from: https://www.rivm.nl/bibliotheek/rapporten/2024-0072.pdf

[3] Rijksinstituut voor Volksgezondheid en Milieu (RIVM). Vaccinatiegraad Rijksvaccinatieprogramma Nederland. Verslagjaar 2025. [Vaccination coverage National Immunisation Programme in the Netherlands 2025]. Bilthoven: RIVM; 2025. Dutch. Available from: https://www.rivm.nl/publicaties/vaccinatiegraad-rijksvaccinatieprogramma-nederland-verslagjaar-2025

[4] Monge S Hahné SJ de Melker HE Sanders EA van der Ende A Knol MJ. Effectiveness of the DTPa-HBV-IPV/Hib vaccine against invasive Haemophilus influenzae type b disease in the Netherlands (2003-16): a case-control study. Lancet Infect Dis. 2018;18(7):749-57. 10.1016/S1473-3099(18)30166-X 29752131

[5] Knuf M Haas H Garcia-Corbeira P Turriani E Mukherjee P Janssens W Hexavalent vaccines: What can we learn from head-to-head studies? Vaccine. 2021;39(41):6025-36. 10.1016/j.vaccine.2021.08.086 34531081

[6] Pluijmaekers AJM Steens A Houweling H Rots NY Benschop KSM van Binnendijk RS A literature review and evidence-based evaluation of the Dutch national immunisation schedule yield possibilities for improvements. Vaccine X. 2024;20:100556. 10.1016/j.jvacx.2024.100556 39444596 PMC11497366

[7] Steens A Stanoeva KR Knol MJ Mariman R de Melker HE van Sorge NM. Increase in invasive disease caused by Haemophilus influenzae b, the Netherlands, 2020 to 2021. Euro Surveill. 2021;26(42):2100956. 10.2807/1560-7917.ES.2021.26.42.2100956 PMC8532506 34676819

[8] Brueggemann AB Jansen van Rensburg MJ Shaw D McCarthy ND Jolley KA Maiden MCJ Changes in the incidence of invasive disease due to Streptococcus pneumoniae, Haemophilus influenzae, and Neisseria meningitidis during the COVID-19 pandemic in 26 countries and territories in the Invasive Respiratory Infection Surveillance Initiative: a prospective analysis of surveillance data. Lancet Digit Health. 2021;3(6):e360-70. 10.1016/S2589-7500(21)00077-7 34045002 PMC8166576

[9] Shaw D Abad R Amin-Chowdhury Z Bautista A Bennett D Broughton K Trends in invasive bacterial diseases during the first 2 years of the COVID-19 pandemic: analyses of prospective surveillance data from 30 countries and territories in the IRIS Consortium. Lancet Digit Health. 2023;5(9):e582-93. 10.1016/S2589-7500(23)00108-5 37516557 PMC10914672

[10] Rijksinstituut voor Volksgezondheid en Milieu (RIVM). The National Immunisation Programme in the Netherlands. Surveillance and developments in 2022-2023.; 2023. Available from: 10.21945/RIVM-2023-0330

[11] Altman DG Bland JM. Interaction revisited: the difference between two estimates. BMJ. 2003;326(7382):219. 10.1136/bmj.326.7382.219 12543843 PMC1125071

[12] O’Loughlin RE Edmond K Mangtani P Cohen AL Shetty S Hajjeh R Methodology and measurement of the effectiveness of Haemophilus influenzae type b vaccine: Systematic review. Vaccine. 2010;28(38):6128-36. 10.1016/j.vaccine.2010.06.107 20655402

[13] Ramsay ME McVernon J Andrews NJ Heath PT Slack MP. Estimating Haemophilus influenzae type b vaccine effectiveness in England and Wales by use of the screening method. J Infect Dis. 2003;188(4):481-5. 10.1086/376997 12898433

[14] McVernon J Andrews N Slack MPE Ramsay ME. Risk of vaccine failure after Haemophilus influenzae type b (Hib) combination vaccines with acellular pertussis. Lancet. 2003;361(9368):1521-3. 10.1016/S0140-6736(03)13171-6 12737866

[15] Hong E Terrade A Denizon M Aouiti-Trabelsi M Falguières M Taha MK Haemophilus influenzae type b (Hib) seroprevalence in France: impact of vaccination schedules. BMC Infect Dis. 2021;21(1):715. 10.1186/s12879-021-06440-w 34330228 PMC8325224

[16] Deghmane A-E Taha M-K. Changes in Invasive Neisseria meningitidis and Haemophilus influenzae Infections in France during the COVID-19 Pandemic. Microorganisms. 2022;10(5):907. 10.3390/microorganisms10050907 35630352 PMC9147110

[17] Marques JG Inácio Cunha FM Bajanca-Lavado MP. Haemophilus influenzae Type b Vaccine failure in Portugal: a nationwide multicenter pediatric survey. Pediatr Infect Dis J. 2023;42(9):824-8. 10.1097/INF.0000000000004011 37406244

[18] European Centre for Disease Prevention and Control (ECDC). Surveillance atlas of infectious diseases. Stockholm: ECDC. [Accessed: 12 Aug 2025].Available from: https://atlas.ecdc.europa.eu/public/index.aspx

[19] Slack M Esposito S Haas H Mihalyi A Nissen M Mukherjee P Haemophilus influenzae type b disease in the era of conjugate vaccines: critical factors for successful eradication. Expert Rev Vaccines. 2020;19(10):903-17. 10.1080/14760584.2020.1825948 32962476

[20] Rijksinstituut voor Volksgezondheid en Milieu (RIVM). The National Immunisation Programme in the Netherlands. Surveillance and developments in 2021-2022. Bilthoven: RIVM; 2022. Available from: https://www.rivm.nl/bibliotheek/rapporten/2022-0042.pdf

[21] Bajanca-Lavado MP Cavaco L Fernandes M Touret T Candeias C Simões AS Haemophilus influenzae carriage among healthy children in Portugal, 2015-2019. Microorganisms. 2022;10(10):1964. 10.3390/microorganisms10101964 36296240 PMC9611606

[22] Giufrè M Daprai L Cardines R Bernaschi P Ravà L Accogli M Carriage of Haemophilus influenzae in the oropharynx of young children and molecular epidemiology of the isolates after fifteen years of H. influenzae type b vaccination in Italy. Vaccine. 2015;33(46):6227-34. 10.1016/j.vaccine.2015.09.082 26440924

[23] van Werkhoven CH de Gier B McDonald SA de Melker HE Hahné SJM van den Hof S Informed consent for national registration of COVID-19 vaccination caused information bias of vaccine effectiveness estimates mostly in older adults: a bias correction study. J Clin Epidemiol. 2024;174:111471. 10.1016/j.jclinepi.2024.111471 39032589

