# Supplementary material for: Product- and schedule-specific vaccine effectiveness against invasive Haemophilus influenzae serotype b (Hib) disease, The Netherlands, 2005 to 2023
Source: Euro Surveill. 2026 May 14;31(19):2500699. doi: 10.2807/1560-7917.ES.2026.31.19.2500699 (PMC13179478; doi:10.2807/1560-7917.ES.2026.31.19.2500699)
Supplement: Supplement [file 25-00699_DeGIER_Supplement.pdf]

This supplementary material is hosted by Eurosurveillance as supporting information alongside the article "Product- and schedule-specific vaccine effectiveness against invasive Haemophilus influenzae serotype b (Hib) disease, The Netherlands, 2005 to 2023", on behalf of the authors, who remain responsible for the accuracy and appropriateness of the content. The same standards for ethics, copyright, attributions and permissions as for the article apply. Supplements are not edited by Eurosurveillance and the journal is not responsible for the maintenance of any links or email addresses provided therein.

Suppl. Table S1: Hib-containing vaccine product and schedule used in the National Immunisation Programme (NIP) for 2004-2023 birth cohorts, The Netherlands

Adapted from RIVM. The National Immunisation Programme in the Netherlands. Surveillance and developments in 2023-2024. 2024. *Appendix 3: Overview of vaccine changes in the NIP from 2000*

Born on or after	Product *	Schedule
1 February 2004	Pentavalent I	3+1 (2, 3, 4, and 11 months)
1 February 2005	Pentavalent II	
1 April 2006 – risk groups only	Hexavalent I	
01 August 2007	Option: Pentavalent I or II* (or hexavalent I for risk groups)	
01 August 2009	Pentavalent II (option removed)	
01 August 2011	Hexavalent I	
01 October 2018	Hexavalent II	
01 November 2020	Hexavalent II	Standard 2+1 (3,5, and 11 months) for children whose mothers received a maternal Tdap[¥] vaccination after 22 weeks of pregnancy (as in NIP)<br><br>Others, including some risk groups, should receive 3+1 schedule (2, 3, 5, and 11 months), with the first dose given between 6-9 weeks.

*Products:
Pentavalent I: DT3aP-IPV/Hib (Infanrix® IPV+Hib); Pentavalent II: DT5aP-IPV/Hib (Pediacel®); Hexavalent I: DT3aP-HBV-IPV/Hib (Infanrix® Hexa); Hexavalent II: DT5aP-HBV-IPV-Hib (Vaxelis®)
[¥] Tdap: Tetanus-Diphtheria-Acellular Pertussis Vaccine. Introduced in NIP in December 2019.

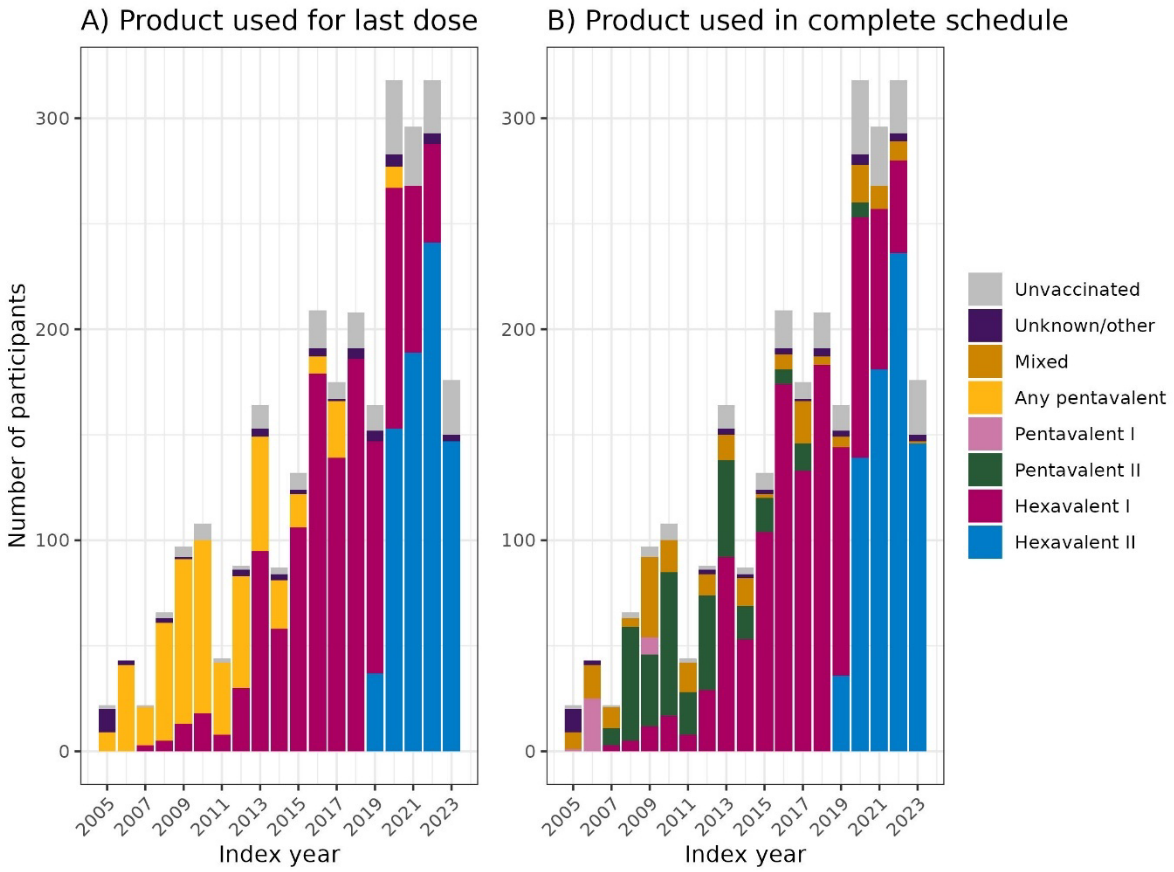

Suppl. Figure S1. Hib containing vaccine product used for the last dose prior the index date (A) and in the complete schedule (B) date by year of index date, The Netherlands, 2005-2023, N=2,737

Suppl. Table S2: Median time since vaccination in days by case status within time since vaccination categories

Time since vaccination	Cases (N=250)			Controls (N=2,487)		
	Count	Percent	Median days (IQR)	Count	Percent	Median days (IQR)
Unvaccinated	113	45.2	-	101	4.1	-
<1 year	51	20.4	166 (142-198)	1,351	54.3	154 (90-198)
1 to <4 years	69	27.6	763 (544-1007)	819	32.9	686 (493-987)
≥ 4 years†	17	6.8	1925 (1673-2496)	216	8.7	1828 (1593-2500)

† Maximum TSV was 9 years (3349 and 3253 days for cases and controls, respectively)

Suppl. Table S3: Median time since vaccination in days for each product and schedule by time since vaccination category. (Note values corresponding to categories where we did not estimate VE are excluded from this table)

Time since vaccination	Exposure	Cases			Controls		
		Count	Percent	Median (IQR)	Count	Percent	Median (IQR)
<1 year	Any pentavalent	16	32.7	172 (143-198)	221	16.7	158 (95-188)
	Hexavalent I	20	40.8	175 (129-203)	541	40.8	156 (98-207)
	Hexavalent II	13	26.5	164 (148-196)	563	42.5	146 (79-199)
1 to <4 years	Any pentavalent	13	18.8	813 (532-1139)	169	21.2	691 (462-1135)
	Hexavalent I	39	56.5	895 (640-1104)	455	57.0	811 (570-1085)
	Hexavalent II	17	24.6	561 (422-700)	174	21.8	509 (426-698)
≥ 4 years	Any pentavalent	7	43.8	2298 (1712-2874)	83	39.7	1781 (1592-2992)
	Hexavalent I	9	56.2	1834 (1673-2009)	126	60.3	1870 (1618-2242)
<1 year	3+1	11	21.6	256 (169-322)	435	33.1	172 (76-265)
	2+1	3	5.9	179 (172-230)	148	11.3	172 (70-264)
1 to <4 years	3+1	59	86.8	798 (538-1046)	729	89.6	708 (504-1013)
	2+1	6	8.8	737 (596-891)	77	9.5	519 (427-710)
≥ 4 years	3+1	16	94.1	1880 (1666-2348)	210	97.7	1828 (1593-2509)

Suppl. Table S4. Number and proportion of vaccinated cases and controls (matched on birth date and sex) and crude vaccine effectiveness estimates against invasive Hib disease, 2005-2023, The Netherlands

Exposure	Time since vaccination	Cases		Controls		Matched Odds Ratio (mOR)	Vaccine effectiveness (VE)	VE 95%CI
		Number (n)	Percent (%)	Number (n)	Percent (%)			
Children aged ≥11-119 months (eligible for full schedule)		**N=172**		**N=1,711**				
Unvaccinated		72	41.9	58	3.4	Ref	Ref	
Fully vaccinated	Any*	100	58.1	1653	96.6	0.043	95.7	93.1-97.3
	<1 year	14	8.3	588	35.3	0.014	98.6	96.6-99.5
	1 to <4 years	65	38.7	809	48.5	0.102	89.8	80.1-94.8
	≥ 4 years	17	10.1	212	12.7	0.063	93.7	70.7-98.6
**Last vaccination product**								
Any pentavalent	<4 years	18	11.9	244	16.9	0.042	95.8	88.2-98.5
	<1 year	5	3	76	4.7	0.123	87.7	44.1-97.3
	1 to <4 years	13	7.8	168	10.3	0.054	94.6	81.5-98.4
	≥ 4 years	7	4.2	83	5.1	0.059	94.1	57.1-99.2
Hexavalent I	<4 years	42	27.8	693	47.9	0.048	95.2	91.2-97.3
	<1 year	3	1.8	240	14.7	0.009	99.1	95.9-99.8
	1 to <4 years	39	23.5	453	27.8	0.118	88.2	75.1-94.4
	≥ 4 years	9	5.4	124	7.6	0.066	93.4	69.2-98.6
Hexavalent II	<4 years	18	11.9	430	29.7	0.019	98.1	94.6-99.3
	<1 year	5	3	261	16	0.002	99.8	96.7-100
	1 to <4 years	13	7.8	169	10.4	0.093	90.7	60-97.9
	≥ 4 years	-	-	-	-	-	-	-
**Schedule**								
2+1	<4 years	9	6	225	15.6	0.027	97.3	93.2-99.0
	<1 year	3	1.8	148	8.9	0.006	99.4	96.1-99.9

Exposure	Time since vaccination	Cases		Controls		Matched Odds Ratio (mOR)	Vaccine effectiveness (VE)	VE 95%CI
		Number (n)	Percent (%)	Number (n)	Percent (%)			
	1 to <4 years	6	3.6	77	4.6	0.108	89.2	64.2-96.7
	≥ 4 years	-	-	-	-	-	-	-
3+1	<4 years	70	46.4	1163	80.4	0.039	96.1	93.3-97.7
	<1 year	11	6.5	434	26.2	0.016	98.4	95.8-99.4
	1 to <4 years	59	35.1	729	44	0.102	89.8	79.8-94.8
	≥ 4 years	16	9.5	210	12.7	0.062	93.8	70.8-98.7
Children aged 6-10 months (eligible for primary series)		**N= 74**		**N= 708**				
Unvaccinated		41	55.4	43	6.1	Ref	Ref	
Fully vaccinated		33	44.6	665	93.9	0.034	96.6	92.9-98.4
**Last vaccination product [¥]**								
Any pentavalent		11	14.9	142	20.1	0.035	96.5	83.4-99.3
Hexavalent I		15	20.3	274	38.8	0.026	97.4	91.1-99.2
Hexavalent II		6	8.1	231	32.7	0.038	96.2	89.8-98.6
**Schedule**								
2+0		5	6.8	154	21.8	0.04	95.9	88.3-98.6
3+0		28	37.8	510	72.1	0.03	96.9	92.7-98.7

*Overall VE includes all children considered fully vaccinated by the NIP at their index date.

Suppl. Table S5. Number and proportion of vaccinated cases and controls and vaccine effectiveness estimates against invasive Hib disease by product used for the complete vaccination schedule, 2005-2023, The Netherlands

Exposure	Time since vaccination	Cases		Controls		Matched Odds Ratio (mOR)	Vaccine effectiveness (VE)	95%CI
		Number	Percent (%)	Number	Percent (%)			
Children aged ≥11-119 months (eligible for full schedule)		**N=173**		**N=1717**				
Unvaccinated		72	47.7	58	4.1	Ref	Ref	
Pentavalent I	<4 years	0	0.0%	0	0.0%	-	-	-
Pentavalent II	<4 years	12	7.9	179	12.5	0.032	96.8	90.6-98.9
	<1 year	1	0.6	41	2.5	0.015	98.5	79.7-99.9
	1 to <4 years	11	6.5	138	8.4	0.065	93.5	77.3-98.1
	≥ 4 years	5	3	55	3.4	0.05	95.0	57.4-99.4
Hexavalent I	<4 years	40	26.5	677	47.3	0.049	95.1	91.0-97.3
	<1 year	2	1.2	233	14.2	0.005	99.5	96.0-99.9
	1 to <4 years	38	22.6	444	27.1	0.124	87.6	73.6-94.1
	≥ 4 years	8	4.8	114	7	0.061	93.9	70.0-98.7
Hexavalent II	<4 years	17	11.3	406	28.4	0.016	98.4	94.9-99.5
	<1 year	5	3	247	15.1	0.008	99.2	95.9-99.8
	1 to <4 years	12	7.1	159	9.7	0.073	92.7	63.1-98.5
	≥ 4 years	0	0.0%	0	0.0%	-	-	-
Mixed	<4 years	10	6.6	111	7.8	0.056	94.4	85.6-97.8
	<1 year	6	3.6	58	3.5	0.096	90.4	66.3-97.3
	1 to <4 years	4	2.4	53	3.2	0.077	92.3	70.3-98.0
	≥ 4 years	4	2.4	40	2.4	0.07	93.0	48.8-99.0
Children 6-10 months (eligible for primary schedule)		**N=74**		**N=698**				
Unvaccinated		41	56.2	43	6.2	Ref	Ref	-
Pentavalent I		3	4.1	31	4.5	0.101	89.9	-3.5-99.0
Pentavalent II		6	8.2	73	10.5	0.037	96.3	74.4-99.5

Hexavalent I	15	20.5	269	38.9	0.028	97.2	90.6-99.2
Hexavalent II	6	8.2	230	33.2	0.038	96.2	89.7-98.6
Mixed	2	2.7	46	6.6	0.034	96.6	77.9-99.5
